# A New Approach to the Quantification of Fibroblast Growth Factor 23—An Array Surface Plasmon Resonance Imaging Biosensor

**DOI:** 10.3390/ijms242015327

**Published:** 2023-10-18

**Authors:** Anna Tokarzewicz, Łukasz Ołdak, Grzegorz Młynarczyk, Urszula Klekotka, Ewa Gorodkiewicz

**Affiliations:** 1 Department of Medical Biochemistry, Medical University of Bialystok, A. Mickiewicza 2C St., 15-089 Bialystok, Poland; 2 Bioanalysis Laboratory, Faculty of Chemistry, University of Bialystok, Ciolkowskiego 1K St., 15-245 Bialystok, Poland; l.oldak@uwb.edu.pl (Ł.O.); ewka@uwb.edu.pl (E.G.); 3 Department of Urology, Medical University of Bialystok, M. Sklodowskiej-Curie 24A St., 15-276 Bialystok, Poland; mlynarz36@yahoo.pl; 4 Department of Physical Chemistry, Faculty of Chemistry, University of Bialystok, Ciolkowskiego 1K St., 15-245 Bialystok, Poland; u.klekotka@uwb.edu.pl

**Keywords:** FGF23, fibroblast growth factor, surface plasmon resonance imaging, biosensors

## Abstract

A new biosensor based on the “surface plasmon resonance imaging (SPRi)” detection technique for the quantification of “fibroblast growth factor 23 (FGF23)” has been developed. FGF23 is mainly produced in bone tissues as a phosphaturic hormone that forms a trimeric complex with “fibroblast growth factor receptor 1 (FGFR1)” and αKlotho upon secretion. FGF23 stimulates phosphate excretion and inhibits the formation of active vitamin D in the kidneys. FGF23 has been shown to play a role in bone carcinogenesis and metastasis. The newly developed method, based on the array SPRi biosensor, was validated—the precision, accuracy, and selectivity were acceptable, and yielded less than ±10% recovery. The rectilinear response of the biosensor ranges from 1 to 75 pg/mL. The limit of detection was 0.033 pg/mL, and the limit of quantification was 0.107 pg/mL. The biosensor was used to determine FGF23 concentrations in the blood plasma of healthy subjects and patients with “clear cell” renal cell carcinoma (ccRCC). The obtained results were compared with those measured through an “enzyme-linked immunosorbent assay (ELISA)”. The determined Pearson correlation coefficients were 0.994 and 0.989, demonstrating that the newly developed biosensor can be used as a competitive method for the ELISA.

## 1. Introduction

Fibroblast growth factor 23 (M = 26 kDa) belongs to the fibroblast growth factor family. These factors are mainly produced by macrophages and are involved in various processes related to normal cell development. They exhibit mitogenic, endocrine, morphological, and regulatory effects. Any abnormality in their function causes developmental defects [1,2].

Osteocytes and osteoblasts produce FGF23 in an inactive form. Its conversion to the active form is accomplished by cutting off the N-terminal fragment from the inactive molecule. The resulting “mature” FGF23 molecule (iFGF23) can be secreted into the blood or degraded intracellularly into two metabolically inactive peptides: the N-terminal and the C-terminal. Because FGF23 can be released into the blood, affecting the function of many tissues and organs, it is called the hormone phosphatonin [3]. In addition to FGF23, the endocrine FGF group includes two other members, i.e., “fibroblast growth factor 19 (FGF19)” and “fibroblast growth factor 21 (FGF21)” [4].

FGF23 is mainly responsible for maintaining the metabolic balance of phosphate and vitamin D. However, this factor is also involved in bone metabolism, iron metabolism, erythropoiesis, the development of inflammatory processes, insulin resistance in tissues, and left ventricular hypertrophy. In recent years, there have been an increasing number of reports on the role of FGF23 in “acute kidney injury (AKI)” [5,6]. FGF23 binds to target cells via the FGFR1. However, the presence of the αKlotho protein co-receptor is also required. Together, these molecules form a three-component complex that initiates signal transduction to the target cell [6,7,8].

A newly developed biosensor was used to measure FGF23 concentrations in patients with clear cell renal cell carcinoma (ccRCC) and healthy subjects in blood plasma samples. ccRCC is the most common histopathological type of renal cell carcinoma. This type of cancer accounts for more than 90% of all renal malignancies [7].

Currently, in the scientific world, only two assays exist that offer the possibility to quantify FGF23 in biological samples. These are the ELISA and “chemiluminescent immunoassay (CLEIA)”. The performance and characteristics of these two methods are fully described in Heijboer et al. [8]. Both of these techniques use special labels that bind to the target molecule, which can affect the obtained results, generating measurement errors. Therefore, it is very important to develop a new label-free method (the array SPRi biosensor), which will significantly expand the field of work for researchers from different scientific disciplines.

During the development of the array FGF23 biosensor, surface plasmon resonance imaging was used as a detection technique. The technique is based on measurements of the SPR signal, which changes as the mass increases due to the binding of successive layers on the chip’s surface. The SPRi technique converts the obtained SPR signal into images that are used as the basis for determining the final SPRi signal. The basics of the SPR and SPRi phenomenon as a method for quantifying various types of analytes were described in an article by Wang et al. [9]. Examples of SPR/SPRi biosensors and their application are described in [10,11,12,13,14,15,16].

The chip architecture and measurement performance of the SPRi array differ significantly from those used in the SPR or SPRi fluidic version. In the fluidic SPRi, the biosensor is created in situ, while in the array SPRi, it is created ex situ. In the fluidic SPRi, the measurement is performed in the presence of the processing solution, while in the array SPRi, it is performed after the removal of this fluid. These differences make the array SPRi more sensitive than the fluidic SPRi. Moreover, concentrations measured with the array SPRi without pre-concentration or signal enhancement require this approach (e.g., signal amplification by using gold nanoparticles) in the fluidic SPRi or the SPR. In addition, the array SPRi allows for the performance the measurements in several samples (usually nine) simultaneously. A series of articles have confirmed the utility of array SPRi biosensors [17,18,19].

To properly perform SPRi measurements, it is necessary to immobilize the compound to be detected (in this study, FGF23) on the surface of the chip. For this purpose, two types of immobilization can be used. The first is based on antigen–antibody interactions [18], while the second is based on antigen–inhibitor interaction [19]. During the development of the FGF23 biosensor, an antigen–antibody type of immobilization was used. A monoclonal mouse anti-human antibody that interacted with FGF23 was used as an antibody.

In this study, cysteamine was used as the linker that is necessary for the binding of the FGF23 antibody to bare gold. This was due to the presence of thiol (SH-) and amine (NH2) groups at both ends of the cysteamine. The thiol group interacted with the bare gold layer to form a self-organized cysteamine monolayer on the chip surface. In further steps of the experiments, this monolayer was used to bind the FGF23 antibody, which was caused by the interaction of the free amine group with the carboxyl group of the antibody. This led to the formation of a covalent amide bond and ultimately enabled the binding of FGF23.

At certain stage of this study, it was noticed that the generated SPRi signal was low and the obtained rectilinear range of the calibration curve was narrow. This was due to the fact that the molecular weight of FGF23 is relatively low. Therefore, it was decided to solve this problem in a way that had not been used before in the development of array SPRi biosensors, namely, by adding a molecule that is found naturally in the human body—the αKlotho protein (M = 110 kDa). This protein is a well-known co-receptor, necessary for FGF23 to bind to the FGFR1. When added to the tested samples, the αKlotho protein bound to the binding region, located at the C-terminal FGF23 fragment, causing the formation of a two-component complex and, at the same time, an increase in the mass of quantified FGF23 [20]. This approach allowed us to enhance the SPRi signal and extend the rectilinear range of the calibration curve, which was used to determine the concentration of FGF23.

The measurements described in this paper were aimed at developing a new array SPRi biosensor that can be used as a new method for quantitative measurements of FGF23. For this purpose, antigen–antibody immobilization was used and the analytical parameters of the biosensor were validated. Also, the utility of the biosensor was tested by comparing the results of the FGF23 concentration measurements that were performed using it with those obtained via an ELISA.

## 2. Results

### 2.1. Selection of the Optimal FGF23 Antibody Concentration

The first step during the development of the FGF23 biosensor was the selection of the optimal concentration of FGF23 antibodies that ensured the complete saturation of the biosensor surface. For this purpose, nine samples with different concentrations of FGF23 antibodies in PBS buffer, i.e., 0.2; 0.5; 1.0; 2.0; 5.0; 10.0; 25.0; 50.0; and 75.0 ng/mL, and a PBS sample, were prepared and applied to the chip with the pre-coated cysteamine monolayer. The volume of each used sample was 2.5 µL. The chip, prepared in such a way, was incubated and cleaned as described in Section 4.3. A mixture of FGF23 and αKlotho (40:169.2 pg/mL) was then applied to the active sites of the biosensor. The purpose of use and the method for selecting and preparing this mixture are described in Section 2.3. After 10 min of the interaction of the antibody with the FGF23-αKlotho complex, the biosensor was washed three times with HBS-ES buffer and five times with distilled water, and then dried. The SPRi signal for all samples was then determined.

**Figure 1:**
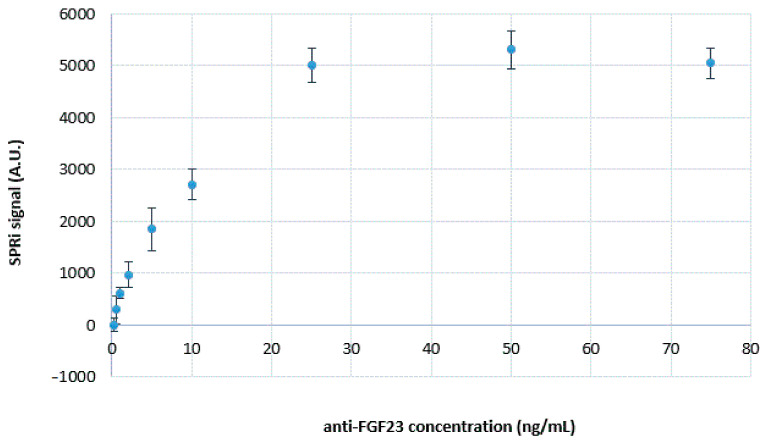
Dependence of the SPRi signal on FGF23 antibody concentration. The used concentration of the FGF23-αKlotho complex was 40:169.2 pg/mL; pH = 7.4. “Standard deviation (SD)” was calculated from 12 individual measurements.

The results of this part of the experiment are shown in Figure 1, where the relation between the SPRi signal and the concentration of FGF23 antibodies is drawn. The obtained SPRi signal was reduced by the PBS buffer signal.

Based on Figure 1, it can be observed that the plateau of this curve began at an FGF23 antibody concentration of 25 ng/mL. This concentration was chosen as the optimal concentration at which a complete saturation of the chip surface with FGF23 antibodies was observed. Therefore, the FGF23 antibody solution of 25 ng/mL was used in further stages of biosensor development and during its validation.

### 2.2. Calibration Curve—The Biosensor’s Response to the Increasing FGF23 Concentration

**Figure 2:**
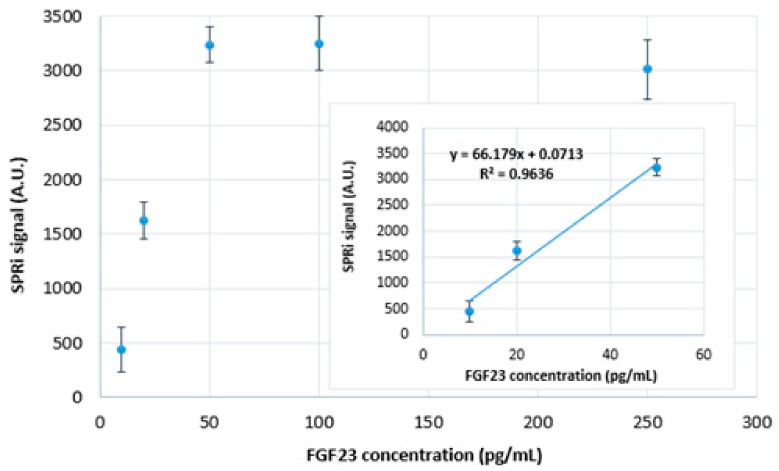
Dependence of the SPRi signal on the FGF23 concentration. The used FGF23 antibody concentration is 25 ng/mL; pH = 7.4. SD was calculated from 12 individual measurements.

The next step in the development of the biosensor was the preparation of a calibration curve, i.e., the response of the biosensor to increasing concentrations of FGF23. For this purpose, a series of samples with different concentrations of FGF23 (2.5 µL each) and PBS samples were applied to the chip with a layer of pre-immobilized FGF23 antibodies (25 ng/mL). The selected and applied FGF23 protein concentrations were 10.0; 20.0; 50.0; 100.0; and 250.0 pg/mL. To allow the FGF23 to interact with the antibodies, the biosensor was left for 10 min. After this process, the biosensor was cleaned according to the technique described in Section 4.3. The rectilinear part of the obtained curve (rectilinear dependence of the SPRi signal on the FGF23 concentration) was used to determine the concentration of FGF23 in the samples. Figure 2 shows the calibration curve and its rectilinear part. The obtained SPRi signal was reduced by the signal of the PBS buffer.

The determined rectilinear response of the biosensor was in the range of 10–50 pg/mL. Due to the fact that FGF23 (M = 26 kDa) is a relatively low-mass molecule, the obtained SPRi signal was low and the determined rectilinearity range of the calibration curve was not satisfactory. Therefore, an attempt was made to enhance and increase the SPRi signal by introducing the αKlotho protein (M = 110 kDa). The use of this protein, which increased the detected mass by binding to FGF23, increased and enhanced the SPRi signal and therefore extended the rectilinear range of the calibration curve.

**Figure 3:**
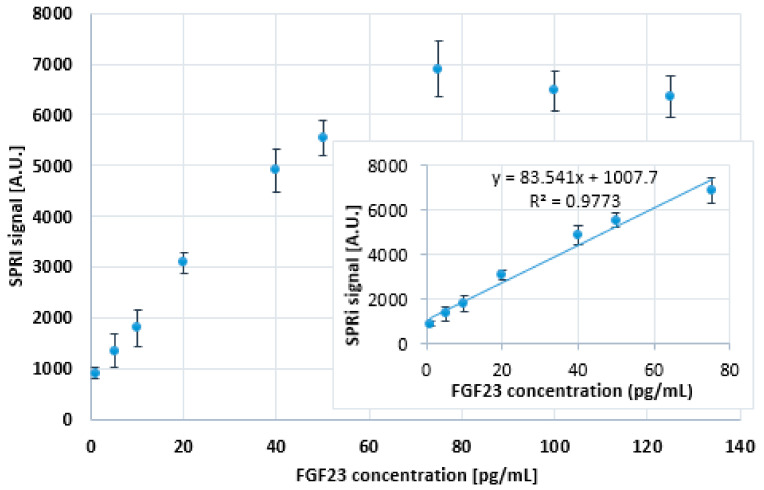
Dependence of the SPRi signal on the FGF23 concentration with the addition of αKlotho protein. The used FGF23 antibody concentration is 25 ng/mL; pH = 7.4. SD was calculated from 12 individual measurements.

For this purpose, nine samples were prepared with different concentrations of FGF23, i.e., 1.0; 5.0; 10.0; 20.0; 40.0; 50.0; 75.0; 100.0; and 125.0 pg/mL. The αKlotho protein was also added to the samples. This protein bound to the FGF23 and formed a much heavier complex than the free FGF23, which was then detected. The mass ratio of the mixture prepared in such a way was 1:4.23 (FGF23/αKlotho). The calibration curve and its rectilinear range obtained after the addition of αKlotho are shown in Figure 3. The SPRi signal was reduced by the PBS buffer signal.

The determined rectilinear response of the biosensor with the addition of αKlotho was broader than without this protein and ranged from 1 to 75 pg/mL. This gave the possibility of determining FGF23 in samples where the concentration was higher than 50 pg/mL. This was also the reason for adding the αKlotho protein to the samples in further parts of this study.

### 2.3. Selection of the αKlotho Concentration and Control of the Complex Creation Time

As mentioned in Section 2.2, during the measurements to obtain a mass ratio of FGF23 to αKlotho protein of 1:4.23, αKlotho protein was added to all tested samples. In determining the appropriate mass ratio, it was assumed that one molecule of FGF23 (26 kDa) binds to one molecule of αKlotho protein (110 kDa) and forms the detected complex. To test the validity of this assumption, a measurement was carried out in which six different samples with different concentrations of both FGF23 and αKlotho protein (also in excess) were prepared. The following samples were used in this part of the experiment (the FGF23 concentration is given first): 5:21.5; 5:42.3; 50:215.0; 50:423.0; 100:423.0; and 100:846.0 pg/mL. The obtained results of the FGF23 concentration measurements are shown in Table 1.

Based on the results shown in Table 1, it can be concluded that no effect of excess αKlotho protein on the FGF23 concentration measurements was observed (recovery less than ±7%). Therefore, in all performed measurements in which the FGF23 concentration was determined, αKlotho protein was added to the samples at a mass ratio of 1:4.23.

To control the time required for the formation of the FGF23–αKlotho complex, four samples with different concentrations of FGF23 (1.0; 10.0; 50.0; and 100.0 pg/mL) were prepared. An appropriate amount of αKlotho (mass ratio 1:4.23) was then added to the samples. The concentration of FGF23 was determined immediately (0 min) and 30 min after the addition of this compound to the samples. The results obtained are shown in Table 2.

The results shown in Table 2 allow us to conclude that no difference between the determined concentrations of FGF23 in the samples applied to the biosensor at 0 and after 30 min was observed. The complex of FGF23 with αKlotho was formed immediately after mixing the two components. Therefore, the samples were applied to the biosensor immediately after mixing the two components.

### 2.4. Selectivity of the Biosensor

The newly developed biosensor was tested for selectivity. Based on the available literature, three potential interfering factors were selected. These were FGF19, FGF21, and albumin. Both FGF molecules were chosen because they are classified in the same family of endocrine FGFs [4] as FGF23. In turn, albumin was chosen for the experiment because it is the protein that is most abundant in the blood plasma—about 60% of the total blood plasma protein mass [21].

Twelve different mixtures (samples) were prepared, which contained FGF23 (40 pg/mL) and interferents with increasing concentration ratios, i.e., 1:1 (40:40 pg/mL), 1:10 (40:400 pg/mL); 1:100 (40:4 ng/mL); and 1:1000 (40:40 ng/mL). The αKlotho protein was also added to the mixtures during their preparation. However, since FGF19 and FGF21 also bind to this protein, the Klotho protein was added in a four-fold excess (1:16.92 instead of 1:4.23) to correctly determine the concentration of FGF23.

Then, in the prepared mixtures, taking into account the SPRi signal of the PBS buffer, the concentration of FGF23 was determined. The results of this part of the experiment are shown in Table 3 below.

The determined recovery of the FGF23 concentration for all tested samples with interferents was less than ±10%. The effect of interferents on the measurements performed was insignificant, and the developed biosensor showed a high selectivity towards the mentioned interferents.

### 2.5. Precision, Accuracy, Detection, and Quantification Limits of the Biosensor

To determine the precision and accuracy of the newly developed biosensor, three different samples (concentrations) of the FGF23–Klotho (1:4.23) mixture were applied to the biosensor, i.e., 1.0 (the lowest concentration); 40.0 (the middle concentration); and 75.0 pg/mL (the highest concentration of the rectilinear range of the calibration curve). Each was applied to three active sites of the biosensor, resulting in 36 replicates for each concentration. The FGF23 concentration, its standard deviation, recovery (a measure of accuracy), and “relative standard deviation (RSD)”, which was a measure of precision, were then determined. All the results obtained from this part of the experiment are collected and presented in Table 4.

The determined recovery of the tested concentration was less than ±5%, indicating that the accuracy of the newly developed method was high. The determined relative standard deviation, a measure of precision, increased with the increasing concentration. For a value of 40 pg/mL, a low and acceptable value for this parameter was obtained.

The next step in the method validation was to determine the “limit of detection (LOD)” and “limit of quantification (LOQ)”. For this purpose, PBS buffer (pH = 7.4, blank sample) was applied to three active sites of the biosensor (12 × 3 = 36 sites). After 10 min of FGF23 interaction with the antibody, the biosensor was cleaned (Section 4.3). The concentration of FGF23 was then determined. The detection limit was calculated based on the following equation:

LOD = 3 × SD/a = 0.033 pg/mL(1)

in which “a” was the directional coefficient of the slope of the calibration curve (83.541). Its value was 0.033 pg/mL.

The limit of quantification was calculated based on the following equation:

LOQ = 10 × SD/a = 0.107 pg/mL(2)

and its value was 0.107 pg/mL.

### 2.6. Recovery, Precision, and Selectivity of the Biosensor Tested in a Biological Sample

In addition, some analytical parameters, such as the recovery, precision, and selectivity (effect of sample matrix background) of the FGF23 concentration measurements, were tested in a biological sample. For this purpose, a mixture of FGF23-αKlotho (1:16.92) was added to a blood plasma sample in which the FGF23 concentration was determined five times before (C_0_) such that the added FGF23 concentration was 25 pg/mL. Since biological samples may have contained other αKlotho protein binding compounds, such as FGF19 and FGF21, it was decided to increase the amount of αKlotho protein added to the sample. In addition, the final concentration (C_found_) of FGF23 in this sample was measured five times, taking into account the signal of the PBS buffer. The FGF23 C_0_ and C_found_ concentrations were determined from 12 individual measurements for each of the five measurements. Based on the measurements, the SDs were calculated for both the C_0_ and C_found_. In addition, the RSD for C_found_ was calculated.

The results shown in Table 5 indicate that the biological sample matrix showed almost zero effect on the FGF23 concentration measurements carried out with the array SPRi biosensor. The determined recovery of C_found_ in all samples was less than ±5%, and the RSD of C_found_ was less than 10%. These results were consistent with those obtained in Section 2.4, and once again confirmed the high selectivity of the developed biosensor towards potential interferents.

### 2.7. Scanning Electron Microscope Measurements

“Scanning electron microscope (SEM)” measurements were carried out to control the formation of individual different layers on the surface of the biosensor, i.e., bare gold, cysteamine, FGF23 antibodies, and the complex of FGF23 with αKlotho protein.

**Figure 4:**
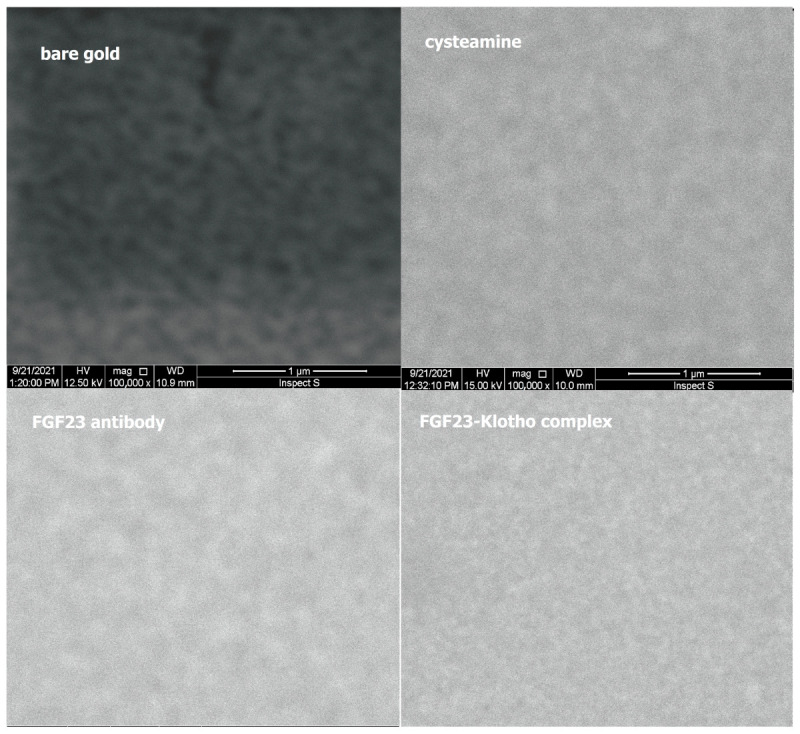
Formation of the FGF23 SPRi biosensor layers, i.e., bare gold, cysteamine, FGF23 antibody, and FGF23-αKlotho complex, captured via SEM. Used parameters: 12.5 or 15 kV, and 100,000× magnification.

The SEM measurements were performed using an INSPEC S60 microscope from FEI, equipped with a tungsten electron source. A voltage of 12.5 or 15 kV and a backscattered electron detector were used for the tests. The samples were placed on aluminum tables and attached with carbon conductive tape for better conductivity. A magnification of 100,000× was used. After each applied layer, the biosensor was washed as described in Section 4.3. The images obtained for each biosensor layer using SEM are shown in Figure 4 below.

Based on Figure 4, it can be seen that each of the successively applied layers on the biosensor showed a different texture. Moreover, each of these layers smoothed and unified the bare gold. Observations of this process confirmed the formation of successive layers on the biosensor.

### 2.8. Confirmation of the SPRi Biosensor Utility

The final step in the development of the SPRI FGF23 biosensor was to confirm its utility by comparing the results obtained using it with those obtained from the ELISA. For this purpose, the FGF23 concentrations were measured in blood plasma samples of healthy subjects and cancer patients using both the SPRI biosensor and the ELISA. The ELISA (Immunotopics, cat. No 60-6600, San Diego, CA, USA) was performed according to the manufacturer’s procedure. The SPRi biosensor was prepared correctly and cleaned after each step of the experiment (Section 4.3). The PBS buffer signal was taken into account. Moreover, since αKlotho protein binding compounds may have been present in the biological samples, the protein was added to the samples in excess (1:16.92). The results obtained using the two techniques and their comparison are summarized and presented in Table 6 below.

**Figure 5:**
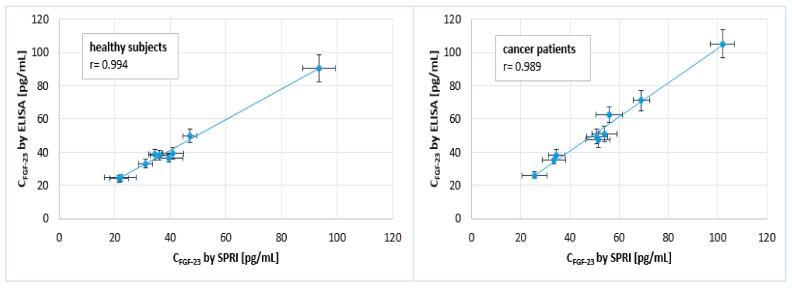
The rectilinear correlation between the two methods, the SPRi biosensor and ELISA. Pearson coefficients were calculated for blood plasma samples of healthy subjects and cancer patients. SD was calculated based on 12 measurements for the SPRi biosensor and 2 for the ELISA.

In addition, a “Pearson correlation coefficient (r)” was calculated based on the obtained results. In general, the coefficient reflected the rectilinear correlation between two variables, which in this experiment were the two methods used to determine the FGF23 concentrations—an ELISA and the newly developed array SPRi biosensor. The coefficients were determined separately for the two types of biological samples; the correlations are shown in Figure 5.

The difference between the determined FGF23 concentrations in the two types of biological samples using an ELISA and the SPRi biosensor was less than ±10% in almost all samples. Pearson correlation coefficients, determined for the two used methods and separately for the two types of biological samples, were close to 1, i.e., 0.994 and 0.989 for the blood plasma of healthy subjects and the blood plasma of cancer patients, respectively. These coefficients indicated that there was a strong positive correlation between the ELISA and the SPRi biosensor. This indicated that the newly developed biosensor gave very similar results for measuring FGF23 concentrations in biological samples to those of an ELISA. Thus, it can be used interchangeably with an ELISA for the quantification of FGF23.

## 3. Discussion

The experiments described in this article aimed to develop a new label-free and optical method for measuring the concentration of FGF23 in biological samples. For this purpose, an array biosensor based on the version of the surface plasmon resonance using imaging—SPRi—was developed.

A new approach used in this work was the enhancement of the analytical SPRi signal by adding a protein that is found naturally in the human body—the αKlotho protein—which significantly improved the analytical characteristics of the biosensor, mainly the range of rectilinearity of the analytical signal.

To properly develop this new method, it was necessary to determine and validate its analytical parameters. For this purpose, a series of different experiments were carried out, which provided the opportunity to determine of optimal concentration of the FGF23 antibodies (25 ng/mL), the rectilinear range of the calibration curve (1–75 pg/mL), the limit of detection (0.033 pg/mL), the limit of quantification (0. 107 pg/mL), the interaction time and concentration of αKlotho protein, and the selectivity (SD < ±10%). A final but very important step in the development of the new method was to confirm its utility by comparing the results of the determined FGF23 concentration using this biosensor with those obtained using the so-called “gold standard”—an ELISA.

In general, ELISAs use special labels that can lead to false-positive or false-negative results by interfering with the normal function of the protein to which they are attached. The newly developed biosensor circumvents this issue and has significant advantages over ELISAs, i.e., measuring FGF23 concentrations is less expensive per sample [22] and consumes much less of a sample (3 µL vs. 50 µL) than ELISAs. This gives the possibility to measure FGF23 concentrations in so-called “unique” samples. A comparison of selected analytical parameters and characteristics of the developed SPRi biosensor and a ELISA (Human FGF23 intact, Quidel-Immutopic, San Diego, CA, USA), made based on the manufacturer’s package insert and the values obtained from the performed measurements, is shown in Table 7.

It is not only the above-mentioned advantages of the biosensor that support its use, but also a comparison of the results of the FGF23 concentration measurements made using the two methods (Table 6). The newly developed array SPRi biosensor showed results that were very similar to those obtained using the ELISA. Pearson coefficients calculated for the two types of biological samples were very close to one, and the difference in the determined concentrations was less than 10%. Based on the parameters shown in Table 7 and the obtained concentration results, it can be concluded that the array SPRI biosensor is suitable and can be used for the accurate determination of FGF23 concentrations interchangeably with ELISAs.

## 4. Materials and Methods

### 4.1. Chemical Reagents, Materials, and Biological Samples

As standards, recombinant human FGF23 protein from a mouse myeloma cell line (M = 26.1 kDa), recombinant human αKlotho protein from a mouse myeloma cell line (109.8 kDa), recombinant human FGF19 from *E. coli* (21 kDa), and recombinant human FGF21 from *E. coli* (20.2 kDa), all from R&D Systems (Minneapolis, MN, USA), as well as albumin from human sera (Sigma-Aldrich, Munich, Germany) were used. The monoclonal mouse anti-human FGF23 antibody (R&D Systems) was a receptor that bound the FGF23 protein from samples to the chip’s surface. As a linker, cysteamine hydrochloride (Sigma-Aldrich) was used.

Additionally, *N*-ethyl-*N*′-(3-dimethylaminopropyl) carbodiimide (EDC), *N*-Hydroxysuccinimide (NHS, all Sigma-Aldrich), absolute ethanol, acetic acid, sodium chloride, sodium acetate (all POCh, Gliwice, Poland), HBS-ES buffer pH = 7.4 (0.01 M HEPES, 0.15 M sodium chloride, 0.005% Tween 20, 3 mM EDTA), and Michaelis phosphate buffer pH = 7.40 (BIOMED, Lublin, Poland) were used as received. Aqueous solutions were prepared with miliQ water (Simplicity^®^MILLIPORE, Merck Milipore, Burlington, MA, USA). High purity (99.999%) argon N 5.0 (AIR LIQUIDE, Białystok, Poland) and a human FGF23 ELISA kit (cat. No 60-6600, Immunotopics, San Diego, CA, USA) were used.

This study was conducted in accordance with the Declaration of Helsinki. It was also approved by the Bioethics Committee of the Medical University of Bialystok (R-I-002/500/2019, 28 November 2019). Informed consent was obtained from all study participants.

All patients’ blood samples used in the study were collected before open nephrectomy surgery and provided by the Department of Urology at the Medical University of Bialystok. Blood samples were also collected from healthy subjects who were honorary blood donors of the Regional Blood Donation Center in Bialystok—the control group. During the blood collection procedure, EDTA was used as an anticoagulant.

After blood collection, two mL of blood was centrifuged (1000× *g*) for 15 min. Next, it was filtered three times to separate the blood plasma from the cells. The blood plasma samples prepared in this way were frozen and stored at −70 °C until further use.

### 4.2. SPRi Apparatus and the SPRi Signal Measurement

All SPRi experiments were performed using a stationary (non-liquid) apparatus developed by the University of Bialystok and AC S.A. The base of each array FGF23 SPRi biosensor was a glass plate coated with a 1 nm titanium layer and a 50 nm gold layer manufactured by Ssens, Enschede, The Netherlands. Individual, successive layers of the linker (cysteamine), FGF23 antibodies, and the tested samples containing a complex of FGF23 with αKlotho were applied to the gold surface of the chip.

The measurement system of the SPRI apparatus consisted of a light source (monochromatic laser diode, λ = 635 nm), which emitted light passing through an array of lenses and polarizers. The emitted light was then reflected from the surface of the biosensor and recorded with a detector—a “CCD camera”. All of the aforementioned elements were mounted on moveable arms, which allowed the angle of incidence of light on the biosensor to be changed. Each of the biosensors used in this study consisted of nine measurement cells, each containing 12 active sites with an appropriately modified gold surface, so that successive layers of the biosensor could be bonded to its surface. A special light-cured polymer separated the cells and the active sites. The diode laser (635 nm) was used to induce surface plasmons during the measurements. The SPRi measurements were carried out in two polarizations of light—p-polarization, which induced the SPR effect, and s-polarization, which suppressed the SPR effect and was used to correct small changes in the intensity of the radiation emitted by the diode.

**Figure 6:**
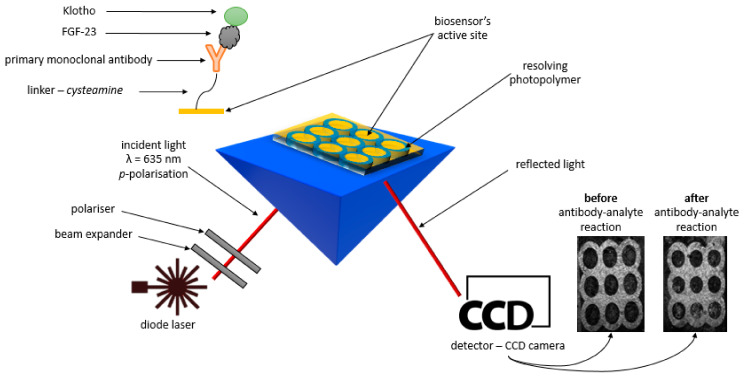
Scheme of the measuring system of the SPRi apparatus and an overview diagram of the FGF23 biosensor.

The final analytical signal was calculated as the difference in the light intensity reflected from the biosensor’s active sites. It was determined from images recorded before and after the interaction of the FGF23 antibody with FGF23, with each corrected by subtracting the background, i.e., the SPR signal at polarity s. Thus, both polarizations of light were used during the measurements. ImageJ software (version 1.51k, National Institutes of Health, Bethesda, MD, USA) was used for the mathematical processing of the obtained images. This procedure for calculating the SPRi signal was used for all performed measurements. A scheme of the SPRi apparatus and the used chips with successively immobilized layers on their surface is shown in Figure 6.

### 4.3. Preparation of the Chip’s Surface for Measurements

Each chip used in the study had to be adequately prepared to be considered an FGF23 biosensor. The process involved creating layers of cysteamine and FGF23 antibodies. For this purpose, chips with a gold layer were immersed at room temperature for at least 12 h in a 20 mM alcoholic solution of cysteamine. Next, the chips with the cysteamine monolayer were washed with absolute ethanol and rinsed with milli-Q water. After cleaning, the chips were dried under an argon atmosphere. The preparation of the chips in the above-described manner allowed them to be stored for a month without any effect of the environment on their properties.

The next step in preparing each chip that was used in the study was to immobilize the FGF23 antibodies. This involved applying a mixture of the activated FGF23 antibodies that, besides the antibodies, contained EDC and NHS (1:1 concentration ratio) in a carbonate buffer solution, which provided an optimal pH = 8.5. After the mixture was applied to the chip, it was incubated in a special incubator for 1 h at 37 °C. After this process, the chip was rinsed 10 times with distilled water and dried.

Additionally, a 1 ng/mL “bovine serum albumin (BSA)” solution was applied on the active sites of each used biosensor with the immobilized antibody layer. After the interaction, the biosensor was washed 10 times with distilled water. This procedure was used to eliminate the nonspecific adsorption. The biosensors prepared in this way were used to determine the concentration of FGF23 in the biological samples.

In addition, at a certain stage of the study (Section 2.2), it was decided to add αKlotho protein to the tested samples. The formed FGF23–αKlotho complex was then bound to the previously immobilized antibody layer on the surface of the biosensor (process described above). After 10 min of interaction, the biosensor was washed three times with HBS-ES buffer and five times with distilled water and dried. Finally, the FGF23 was quantified based on the calibration curve.

Based on the studies described in Section 2.3, it was found that the complex was created immediately after mixing the FGF23 solution/biological sample with the αKlotho protein solution, without the use of any additional procedures, e.g., extended FGF23- αKlotho protein interaction time.

## 5. Conclusions

The results discussed in this publication confirmed the development of a new method for the quantification of FGF23—the array SPRi biosensor. Its utility and applicability were confirmed based on its acceptable precision and accuracy values (SD < ±5%), low detection (0.033 pg/mL) and quantification (0.107 pg/mL) limits, and high selectivity against interferents/the sample matrix (SD < ±10%). In addition, the Pearson coefficients determined for the two groups of biological samples—0.994 and 0.989—showed the positive correlation between the results obtained with the array SPRi biosensor and those obtained with the “gold-standard” ELISA. These results indicated that the newly developed biosensor can be used interchangeably with ELISAs for the quantification of FGF23 in biological samples. This is very important in view of the fact that, to date, only two tests exist that make this possible (ELISA and CLEIA), which significantly limits the possibilities for research.

## Figures and Tables

**Table 1:** Influence of the αKlotho protein on the detected FGF23 concentration. SD was calculated from 12 individual measurements.

Prepared Concentration FGF23/αKlotho (pg/mL)	Detected Concentration of FGF23 (pg/mL)	Recovery (%)	SD (pg/mL)
5:21.5	4.73	94.60	0.73
5:42.3	5.07	101.40	0.82
50:215	52.34	104.68	2.61
50:423	53.28	106.56	4.74
100:423	98.76	98.76	3.28
100:846	103.41	103.41	5.41

**Table 2:** Influence of the time on the formation of the FGF23-αKlotho complex. SD was calculated from 12 individual measurements.

Time of the FGF23 Interaction with αKlotho (min)	The Prepared Concentration of FGF23 and αKlotho Mixture (pg/mL)	Detected Concentration of FGF23 (pg/mL)	Recovery (%)	SD (pg/mL)
0	1:4.23	1.07	107.00	0.54
	10:42.3	10.98	109.80	0.36
	50:211.5	51.65	103.30	0.69
	100:423	102.39	102.39	0.98
30	1:4.23	1.12	112.00	0.39
	10:42.3	11.3	113.00	0.41
	50:211.5	50.58	101.16	0.74
	100:423	103.82	103.82	0.81

**Table 3:** The influence of the potential interferents on the determination of the FGF23 with array SPRi biosensor. SD was calculated from 12 individual measurements.

Interferent	Concentration Ratio	The Determined Concentration of FGF23 (pg/mL)	Recovery (%)	SD (pg/mL)
FGF19	1:1	38.17	95.35	1.93
	1:10	43.69	109.24	2.16
	1:100	37.93	94.84	1.87
	1:1000	37.63	94.08	2.28
FGF21	1:1	38.63	96.62	2.94
	1:10	42.59	106.46	1.54
	1:100	37.67	94.18	2.78
	1:1000	43.25	108.11	1.44
Albumin	1:1	41.09	102.72	1.40
	1:10	41.91	104.79	3.96
	1:100	38.07	95.18	3.04
	1:1000	40.26	100.65	2.37

**Table 4:** Precision and accuracy of the FGF23 SPRi biosensor. SD and RSD were calculated from 36 individual measurements.

The Applied Concentration of FGF23 (pg/mL)	The Determined Concentration of FGF23 (pg/mL)	SD (pg/mL)	Recovery (%)	RSD (%)
1	0.98	0.22	98	22.45
40	41.32	0.63	103	1.52
75	73.65	0.57	98	0.77

**Table 5:** Precision and recovery of the FGF23 SPRi biosensor tested in a plasma sample after the addition of a 25 pg/mL spike. SD and RSD were calculated from 12 individual measurements.

C_0_ ± SD (pg/mL)	C_expected_ (pg/mL)	C_found_ ± SD (pg/mL)	RSD (%)	Recovery (%)
12.35 ± 0.73	37.35	36.67 ± 0.62	94.5	98.2
12.52 ± 0.42	37.52	36.78 ± 0.53	94.1	98.0
12.44 ± 0.55	37.44	35.93 ± 0.87	87.9	96.0
12.11 ± 0.69	37.11	37.92 ± 0.76	106.7	102.2
12.24 ± 0.88	37.24	37.92 ± 0.76	106.3	101.8

**Table 6:** The comparison of the results of FGF23 concentration measurements performed with the SPRi biosensor and ELISA in blood plasma samples. SD was calculated based on 12 measurements for the SPRi biosensor and 2 for the ELISA.

Blood Plasma of Healthy Subjects				Blood Plasma of Cancer Patients			
Sample Number	C_FGF23_ with ELISA (pg/mL)	C_FGF23_ with SPRi Biosensor (pg/mL)	C_FGF23_ with Biosensor/C_FGF23_ with ELISA × 100% (%)	Sample Number	C_FGF23_ with ELISA (pg/mL)	C_FGF23_ with SPRi Biosensor (pg/mL)	C_FGF23_ with Biosensor/C_FGF23_ with ELISA× 100% (%)
1	39.50 ± 2.42	36.59 ± 5.01	92.63	1	54.00 ± 4.59	51.02 ± 5.11	94.48
2	22.00 ± 1.87	24.62 ± 5.68	111.90	2	33.50 ± 2.85	35.55 ± 4.89	106.11
3	31.00 ± 2.64	32.99 ± 2.58	106.41	3	25.50 ± 2.17	26.18 ± 5.06	102.67
4	41.00 ± 3.49	39.14 ± 3.79	95.46	4	51.50 ± 4.38	47.48 ± 4.88	92.19
5	93.50 ± 7.95	90.44 ± 5.90	96.72	5	51.00 ± 4.34	49.42 ± 4.31	96.90
6	21.50 ± 1.83	23.95 ± 3.32	111.40	6	102.00 ± 8.67	105.23 ± 4.88	103.16
7	34.50 ± 2.93	38.48 ± 2.43	111.53	7	69.00 ± 5.87	71.04 ± 3.30	102.96
8	47.00 ± 4.00	49.91 ± 2.48	106.19	8	56.00 ± 4.76	62.72 ± 5.37	112.00
9	36.00 ± 3.06	38.29 ± 3.26	106.36	9	34.50 ± 2.93	38.45 ± 3.33	111.45

**Table 7:** A comparison of selected analytical parameters and characteristics of the array SPRi biosensor and the ELISA.

Parameter/Characteristic	ELISA	SPRi Biosensor
Usage of labels	Yes	No
Reusability	No	Yes
Approximate measurement time (min)	245	120
Number of tested samples	96	9 (each in 12 repetitions)
Volume of the tested sample (mL)	50	3
Limit of detection (pg/mL)	1.500	0.033
Limit of quantification (pg/mL)	Non-available	0.107
Rectilinear range of calibration curve (pg/mL)	22–220	1–75

## Data Availability

All data generated or analyzed during this study are included in this article. Further enquiries can be directed to the corresponding author.
